# Genome Analysis of Mayaro Virus Imported to Germany from French Guiana

**DOI:** 10.3201/eid2007.140043

**Published:** 2014-07

**Authors:** Barbara Friedrich-Jänicke, Petra Emmerich, Dennis Tappe, Stephan Günther, Daniel Cadar, Jonas Schmidt-Chanasit

**Affiliations:** Institute of Tropical Medicine and International Health, Berlin, Germany (B. Friedrich-Jänicke);; Bernhard Nocht Institute for Tropical Medicine, Hamburg, Germany (P. Emmerich, S. Günther, D. Cadar, J. Schmidt-Chanasit);; German Centre for Infection Research, Hamburg (J. Schmidt-Chanasit)

**Keywords:** Mayaro virus, viruses, genome analysis, travel, Germany, French Guiana

**To the Editor:** Mayaro virus (MAYV), a mosquito-borne New World alphavirus of the family *Togaviridae*, causes a febrile arthralgia syndrome resembling dengue and chikungunya fever. The virus is maintained in a natural cycle involving nonhuman primates and *Haemagogus* spp. mosquitoes in tropical rainforest areas of South America (*1*). After an incubation time of 7–12 days following an infectious mosquito bite, rash, fever, headache, and arthralgia develop in patients, followed by restoration to their original conditions after several weeks (*1*).

Outbreaks of Mayaro fever have been reported from the Amazon region (*1**,**2*). There are increasing reports of travel-related infections imported from South America to Europe and the United States (*3**–**7*). We describe an acute MAYV infection in a German traveler who returned from French Guiana. Full-length MAYV genome amplification was performed on virus obtained from a serum sample of the patient.

In August 2013, a 44-year-old woman (bookkeeper) came to an outpatient clinic with fever (temperature ≤38.7°C), chills, a mild headache, severe fatigue, highly painful swelling of small finger joints, and pain in both feet. Symptoms appeared 2 days before when she experienced aches in her wrists and left forefoot. Four days before, the patient had returned from a 2.5-week visit to French Guiana, where she traveled with her partner and caught butterflies. She had conducted these activities during her holidays for the past 5 years, mostly in spring or autumn. In July 2013 at the end of the rainy season, she had many mosquito bites, especially on her hands, despite use of repellents and bed nets.

Physical examination showed a body temperature of 38°C, throat enanthema, generalized macular exanthema, and slightly swollen and tender interphalangeal joints of the hands and feet. Her medical history was unremarkable, and her partner was asymptomatic. Laboratory tests showed reference values for hemoglobin concentration; platelet count; and levels of liver enzymes, creatinine, and anti-nuclear and anti–citrulline peptide antibodies. C-reactive protein level was increased (24.2 mg/L; reference value <5 mg/L), and serum lactate dehydrogenase level was slightly increased (4.4 μkat/L; reference value <4.12 μkat/L). Leukopenia (2.4 G/L; reference value 4.0–10.0 G/L) was present, which intensified the next day (2.0 g/L). The leukocyte count returned to a reference value 8 days after disease onset and the patient fully recovered.

Malaria, dengue fever, and rickettsiosis were excluded by using several tests. Blood cultures obtained on day 2 after disease onset remained sterile, and a viral infection was suspected. Follow-up investigation on day 16 of illness showed an increased IgG titer (80) against chikungunya virus (by indirect immunofluorescence assay; reference value <1:20) (*6*) but no IgM titer. Additional tests for alphaviruses were then performed on the same sample, and indirect immunofluorescence assay showed an IgM titer of 2,560 and an IgG titer of 10,240 (reference value <20) (*6*) against MAYV. Results of serologic tests were negative for Venezuelan equine encephalitis virus, Eastern equine encephalitis virus, and Oropouche virus. IgM (80) and IgG (160) titers for antibodies against Ross River virus were low.

An acute MAYV infection was strongly suspected and a stored serum sample from day 2 underwent generic reverse transcription PCR (RT-PCR) for alphaviruses with primers VIR2052F (5′-TGGCGCTATGATGAAATCTGGAATGTT-3′) and VIR2052R (5′-TACGATGTTGTCGTCGCCGATGAA-3′) (*8*) and quantitative MAYV real-time RT-PCR (in-house) with primers MayaroF (5′-CCTTCACACAGATCAGAC-3′), MayaroR (5′-GCCTGGAAGTACAAAGAA-3′), probe labeled with 6- carboxyfluorescein (FAM) and black hole quencher 1 (BHQ-1) MayaroP (5′-FAM-CATAGACATCCTGATAGACTGCCACC-BHQ1–3′) by using the AgPath-ID One-Step RT-PCR Kit (Life Technologies, Carlsbad, CA, USA) according to the manufacturer’s instructions. The generic RT-PCR for alphaviruses showed a positive result, and direct sequencing of the amplicon showed a MAYV-specific sequence. The serum sample had an MAYV viral load of 1.24 × 10^7^ copies/mL when in vitro–transcribed RNA from a reference plasmid was used as a quantification standard.

Attempts to isolate MAYV in cell culture were not successful. Therefore, the serum sample was used to obtain the complete MAYV genome sequence by using primers designed from multiple alignments of the MAYV genomes obtained from databases. (Primer sequences used are available on request.) The complete MAYV genome (strain BNI-1, KJ013266) was amplified from the serum sample, and phylogenetic analysis of a 2-kb genomic fragment showed that strain BNI-1 belonged to genotype D (*9*) and is closely related to strains circulating in Brazil (Figure, Appendix).

**Figure:**
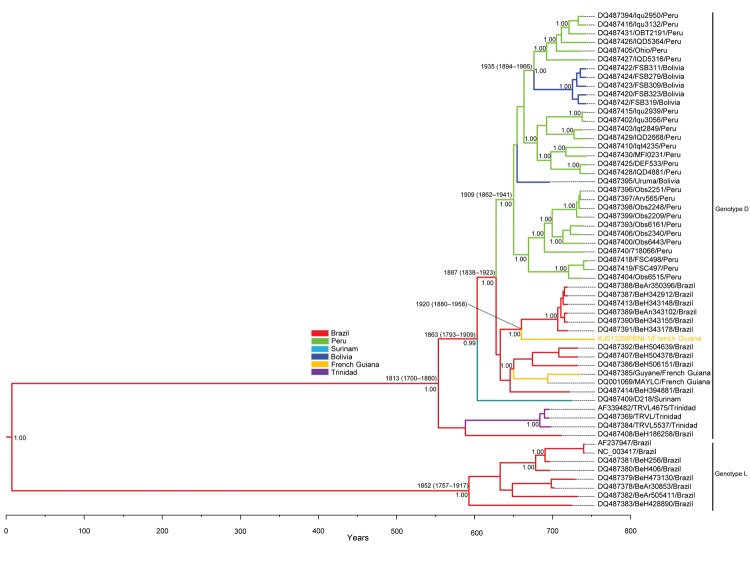
Bayesian maximum clade credibility tree representing the time-scale phylogeny of Mayaro virus (MAYV) by analysis of 2-kb genomic fragments, including the 3′ segment of the envelope (E)2 protein, the complete E1 protein, and the 3′ non-coding region (NCR). Phylogenetic analysis was performed by using Bayesian Markov chain Monte Carlo (MCMC) tree-sampling method implemented in BEAST (http://beast.bio.ed.ac.uk). The general time reversible model of nucleotide substitution with gamma distributed rate variation among sites and a relaxed (uncorrelated log-normal) molecular clock model were used. Two independent runs of 5 × 10^7^ generations with a burn in of 5 × 10^6 ^generations were performed to estimate the posterior probability distribution. Convergence of parameters was confirmed by calculating the effective sample size with Tracer v1.4 (http://tree.bio.ed.ac.uk/software/tracer/) and excluding an initial 10% for each run. The maximum clade credibility tree obtained (tree with the largest product of posterior clade probabilities) was selected from the posterior tree distribution after a 10% burn in by using the TreeAnnotator (http://beast.bio.ed.ac.uk/TreeAnnotator). Taxon information includes GenBank accession number, strain designation, and country of origin. Branches are colored on the basis of the most probable location state of the descendent nodes (see color codes). The posterior probabilities (clade credibilities ≥90%) and the time to most recent common ancestor of the major branches are shown. Date of divergence (95% highest posterior density) of the genotypes, subtypes, and the MAYV strain isolated in this study (in orange) are given. Scale bar indicates years before the last sampling time (2013).

In 2 clinic-based syndromic surveillance studies in South America, 0.8%–3% of febrile episodes were caused by MAYV infection (*2**,**10*). In travelers, MAYV infections were acquired in tropical rainforest or wildlife conservation areas (*7*) and were sometimes associated with insect-hunting activities (*5*). Successful complete genome amplification of MAYV strain BNI-1 from a clinical sample might help identify regions in the MAYV genome that undergo rapid mutations caused by the isolation process in cell culture and improve phylogenetic and functional genome analysis. Moreover, the viral load in our patient was high enough for efficient transmission of MAYV to a susceptible mosquito vector (S. Becker, pers. comm.). Thus, in disease-endemic regions, patients with an acute MAYV infection should be protected from mosquito bites during the first week of disease to prevent spread of the virus.
